# Experimental evaluation of thermal rectification in a ballistic nanobeam with asymmetric mass gradient

**DOI:** 10.1038/s41598-022-11878-2

**Published:** 2022-05-12

**Authors:** Adib Tavakoli, Jeremie Maire, Boris Brisuda, Thierry Crozes, Jean-François Motte, Laurent Saminadayar, Eddy Collin, Olivier Bourgeois

**Affiliations:** Univ. Grenoble Alpes, CNRS, Grenoble INP (Institute of Engineering, Univ. Grenoble Alpes), Institut Néel, 38000 Grenoble, France

**Keywords:** Nanoscale materials, Nanoscale devices, Physics

## Abstract

Practical applications of heat transport control with artificial metamaterials will heavily depend on the realization of thermal diodes/rectifiers, in which thermal conductivity depends on the heat flux direction. Whereas various macroscale implementations have been made experimentally, nanoscales realizations remain challenging and efficient rectification still requires a better fundamental understanding of heat carriers’ transport and nonlinear mechanisms. Here, we propose an experimental realization of a thermal rectifier based on two leads with asymmetric mass gradients separated by a ballistic spacer, as proposed in a recent numerical investigation, and measure its thermal properties electrically with the microbridge technique. We use a Si$_{3}$N$_{4}$ nanobeam on which an asymmetric mass gradient has been engineered and demonstrate that in its current form, this structure does not allow for thermal rectification. We explain this by a combination of too weak asymmetry and non-linearities. Our experimental observations provide important information towards fabricating rigorous thermal rectifiers in the ballistic phonon transport regime, which are expected to open new possibilities for applications in thermal management and quantum thermal devices.

## Introduction

From civilizational challenges such as climate change to recent nanodevices, heat has often been seen as something to be endured due to our lack of control over it. Together with the advancement of our fundamental understanding of thermal transport, the continuous development of nanomaterials and nanodevices brings new possibilities to harvest, control and even make use of heat beyond the traditional Fourier law of heat conduction. Given the extreme societal impact of microelectronics, many works have been dedicated to investigating the physical mechanisms of heat transport in silicon nanostructures^1^, highlighting the importance of geometry, composition, heat carrier scattering, and a range of factors influencing the ability of a structure to transport heat^2,3^. Ultimately, the improved understanding nanoscale heat transport is expected to find broad applications from thermal management to quantum thermal systems, energy harvesting and scavenging, as well as the realization of a self-driven thermal computer.

For thermal management as well as information processing using heat, thermal rectifiers and diodes are key elements that embody the control over thermal transport^4–9^. Thermal diodes are the analogues of electrical diodes for the heat flux, whereas rectifiers represent “imperfect” diodes in which the heat flux is not cut off in one direction but simply reduced. They can for example be used to efficiently dissipate heat from electronic components while protecting them from heat emitted by other components on the chip, or as elementary building blocks for information processing. The general concept of a thermal rectifier, first demonstrated by Starr in 1936^10^, has since been realized following different physical principles, all of which can be controlled for macroscopic structures. One of the most straightforward implementations of a thermal diode is the two-segment one^5^, in which thermal conductivity varies in opposite manners with temperature for the two materials, i.e. thermal conductivity increases with increasing temperature for one material, whereas it decreases for the other. At the nanoscale, thermal properties of nanoscale deviate from their bulk counterpart and thermal rectification can therefore rely on different principles. Mainly, we can distinguish the use of phonon spectra overlap between two regions or materials, the dependence of the thermal conductivity on temperature and geometry/space, local phonon boundary scattering effects as well as asymmetric geometries^11^. Regardless of the scheme chosen, it has been shown that non-linearities together with system asymmetry^4,12–19^ are required for a device to be qualified as a thermal rectifier/diode and that simple ballistic phonon scattering on asymmetric shapes was unlikely to lead to rectification^7^. Nanopatterning can enable artificial thermal metamaterials with local tuning of the physical and thermal properties, such as sonic and phononic crystals, and thus also presents exciting possibilities in thermal rectification. Numerous experimental realizations of such structures have shown that it was possible to exert partial control over the thermal conductivity and its dependence with temperature, via phonon boundary scattering in most conditions^2,3,20–32^ or coherent effects at ultralow temperatures^33,34^. In this context, asymmetric perforated geometries have been proposed as one of the promising ways of making thermal rectifiers at the nano and microscale, and an experimental implementation has been recently demonstrated^35^. In a similar spirit, structures with asymmetric mass gradients would act not on the temperature dependence of thermal conductivity, but on the overlap of phonon spectra originating from either side of the asymmetric structure. Several implementations have been proposed, especially in carbon based materials^36–40^. Recently, Chen at al.^41^ have proposed such a structure, comprising of two leads with asymmetric masses linked by a ballistic spacer, whose function is to make the thermal rectification factor robust against size increases. Although many applications require temperatures at or above room temperature, phonon wavelengths are much larger at ultralow temperatures and ballistic channels can thus be created experimentally at the microscale.

In this work, we propose an experimental realization of that thermal rectifier designed around asymmetric mass gradients linked by a ballistic spacer. We base our structure on a suspended nanobeam that we investigate in the ballistic phonon transport regime, i.e., at temperatures as low as 70 mK. In such structures, a higher heat flux is expected from the heavy to the light side compared to the reverse direction. Our observation shows that no such rectification occurs in this situation. We surmise that rectification in such structures might only be observed in the Ziman regime, i.e., when both ballistic and diffusive transport occurs. We also estimate that a stronger mass gradient might affect the ballistic transport enough to enter a rectification regime. The structure is fabricated in a stoichiometric Si$_{3}$N$_{4}$ layer and monolithically integrated in the measurement device. All measurements are performed at very low temperatures (70 mK to 5 K) in ultra-high vacuum conditions using the microbridge method, where one membrane is Joule heated and the heat flux traversing the structure of interest increases the temperature of the second membrane, which is detected electrically. The measurement system is fully reversible and is therefore ideal to investigate thermal rectification. The results presented in this work provide leads to more efficient realizations of thermal rectification at the nanoscale in nanostructured materials.

## Materials and methods

### Principle of the thermal diode

**Figure 1:**
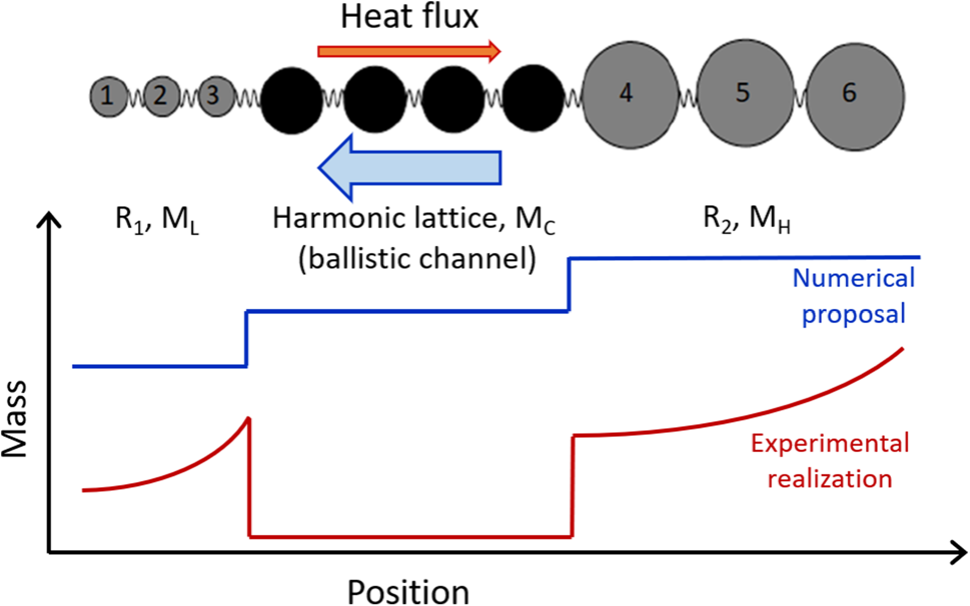
Principle of the asymmetric mass gradient thermal rectifier. (Color online) Schematic principle of the thermal rectifier as suggested by Chen et al. ^41^. It can be modeled by a chain comprising of different masses, linked to two diffusive reservoirs R$_{1}$ and R$_{2}$. Higher heat flux is expected from the heavy (high mass M$_{H}$) to the light side (low mass M$_{L}$) compared to the reverse direction. The relative masses as a function of position are schematically shown for both the numerical proposal (blue) and our experimental implementation (red). The two curves are offset for clarity.

Among the several schemes that have been proposed to make a nanoscale thermal rectifier, we have chosen to focus on the asymmetric mass gradient. The first experimental realization of this scheme was demonstrated by Chang et al.^6^ using carbon and boron nitride nanotubes gradually loaded on one side, in which they demonstrated around 5 and 7% thermal rectification, respectively. A recent work has described the theoretical framework of such thermal rectifiers^41^, more specifically with a structure comprising two non-linear systems coupled to different non-linear on-site potentials by a ballistic thermal channel. Such implementation has been theoretically shown to remain efficient as the system size increases and maintains high rectification factors due to the match or mismatch of phonon bands depending on the heat flux direction. A general schematic of the principle is shown in Fig. 1. We have investigated the impact of asymmetric mass gradients directly inspired by that numerical proposal. Practically, we have chosen a ballistic thermal conductor, which can be described by Landauer formalism, to remove the size dependence of the rectification factor. We expect the absence of losses to increase the rectification factor and previous studies have shown that a higher heat flux is expected from the heavy to the light side than in the reverse direction.

### Experimental realization and characterization

**Figure 2:**
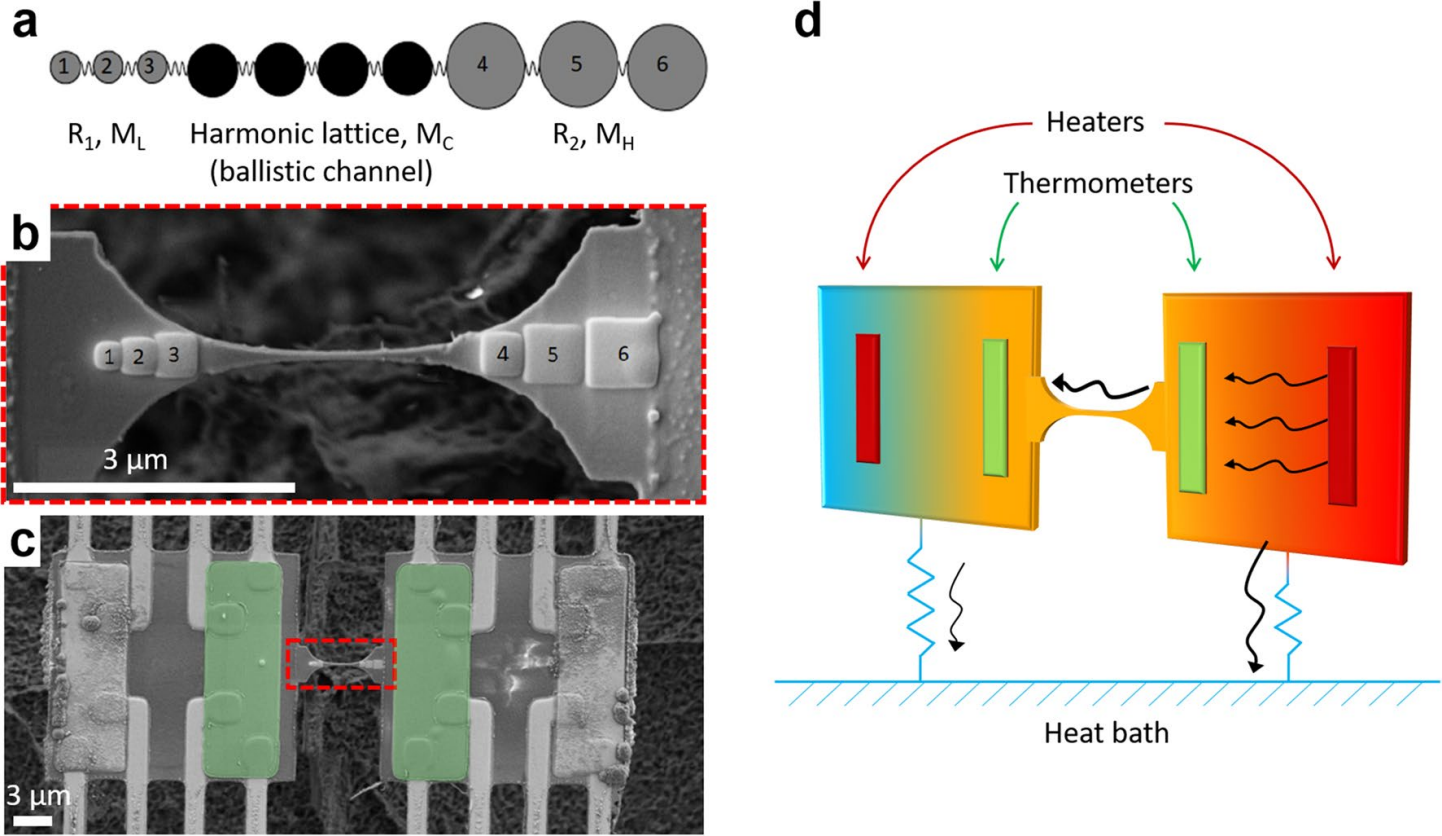
Asymmetric mass gradient nanobeam. (**a**) Schematic plot of the numerical model, in which the two diffusive reservoirs (R1 and R2 ) are linked by the ballistic spacer. (**b**) SEM image of the nanobeam loaded with asymmetric masses. (**c**) False color SEM micrograph of the two suspended membrane-based nanocalorimeters, with the loaded nanobeam from (**b**) between them. The NbN thermometers are represented in green. (**d**) Schematic of the measurement system: the thermometers are shown in green whereas the heaters are shown in red. Heat flow is depicted by black arrows.

The experimental realization of this scheme is based on a 100 nm thick Si$_{3}$N$_{4}$ nanobeam with a central region where phonon transport is ballistic^42^ and connected to the heat baths by two regions of catenoidal shape^43^ designed to minimize phonon scattering and, based on numerical calculations, maximize the transmission coefficient^43,44^. The mass gradients consist of series of square layers of Pt/C deposited by Focused Ion Beam (50% platinum and 50% carbon) with a material density of 11.5 g.m$^{-3}$. The squares sizes, higher on the right side of the ballistic channel, are progressively increased from left to right on each side, i.e., towards and away from the ballistic channel on the left and right side, respectively. The dimensions and masses of deposited materials are presented in Table 1 and the mass distribution is schematically shown in Figs. 1 and 2a. The fabricated structure is shown in Fig. 2b. The mass gradient between the two reservoirs is about $\bigtriangleup$ $m$ =0.48 pg, which is to be compared to the estimated mass of the central nanobeam of $m_{beam}=0.1$ pg.

**Table 1:** Dimensions and masses of deposited material (Pt/C) on each side of the ballistic channel.

	**1**	**2**	**3**	**4**	**5**	**6**
Surface (nm$^2$)	270 $\times$ 270	350 $\times$ 330	400 $\times$ 400	385 $\times$ 400	550 $\times$ 550	680 $\times$ 680
Mass (pg)	0.07	0.10	0.15	0.14	0.27	0.40

The deposited thickness is estimated to be t = 70 ± 30 nm.

The experimental characterization is performed by measuring the temperature-dependent thermal conductance in the temperature range from $\sim$ 70 mK to 5 K, ensuring a ballistic phonon transport in the central part of the nanobeam (the ballistic spacer), as already demonstrated in an anterior work^42^. The structure of interest is suspended between two nominally identical membranes that act as heat reservoirs, the temperature of which can be controlled independently. The measurement platform is shown in Fig. 2c and consists of two adjacent membranes, each suspended by eight supporting beams. Each membrane can be reversibly the heater and sensor, depending on the need. They are thermally connected by the structure under study, which is fabricated monolithically, i.e., in the same 100 nm thick layer of high-stress Si$_{3}$N$_{4}$, limiting any kind of thermal contact resistance. The membranes are drawn by laser lithography, whereas the small size of the central nanobeam requires electron-beam lithography. On each membrane are patterned a copper heater and a niobium nitride (NbN) resistive thermometer^45,46^, highlighted in green in Fig. 2c. This material has a thermal coefficient of resistance of above 1 K$^{-1}$ around 0.1 K. Combined with this high coefficient, a low noise measurement technique allows us to precisely measure the resistance and thus estimate the dissipated power. Our double membrane nanocalorimeter has a state-of-the-art power sensitivity of 15 attoWatt/$\sqrt{\text {Hz}}$ at 0.1 K. The sensitivity on the thermal conductance measurement is thus 1.5$\times$10$^{-16}$ W.K$^{-1}$.$\sqrt{\text {Hz}}$) (0.15 femtoWatt.K$^{-1}$.$\sqrt{\text {Hz}}$))^47^. Measurement is performed in the steady-state, with a continuous heat flow between the two membranes. With its high sensitivity and reversibility—the heat flux can be measured reversibly, i.e. in either direction, due to both reservoirs being nominally identical—this platform is ideally suited for very low temperature measurements and thermal rectification characterization, respectively. The general principle of the measurement is depicted in Fig. 2d.

**Figure 3:**
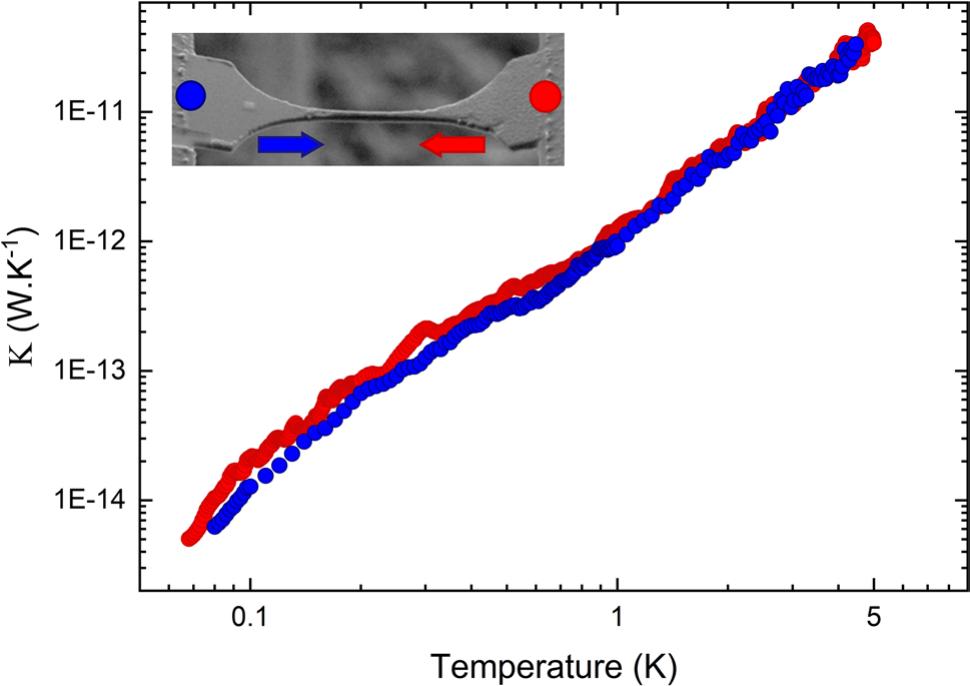
Thermal conductance of the non-loaded nanobeam. Measured thermal conductance of a non-loaded nanobeam linking the membrane-based calorimeters. The measurements are performed alternatively when the left (right) membrane is used as a heater and the other as a sensor. (Upper inset) SEM micrograph of the nanobeams. The dot and arrow of a given color correspond to the heated side and heat flux direction, respectively.

## Results

We first characterize the reciprocity of thermal transport in the non-loaded nanobeam. The experiment is performed with one symmetric nanobeam linking the two suspended membranes. We see in Fig. 3 that the thermal conductance of the nanobeam is the same independently of the heat flux direction in the whole temperature range from $\sim$70 mK to 5 K. In this temperature range, phonon transport in these non-loaded systems is fully ballistic, as has been demonstrated in a previous work^42^. The slight discrepancy in thermal conductance between heat flux directions can be attributed to fabrication inaccuracies. We have also verified that heat transport is independent of the heat flux direction in a system comprising two nominally identical nanobeams in parallel (not shown here). The measurement of the resistance also allows us to extract the temperature difference between the heater and the cold membrane. Due to the ultralow temperatures at which these measurements are performed, we have kept this difference low, between 70 mK when the experiment is performed below 100 mK to around 10 mK at higher temperatures.

**Figure 4:**
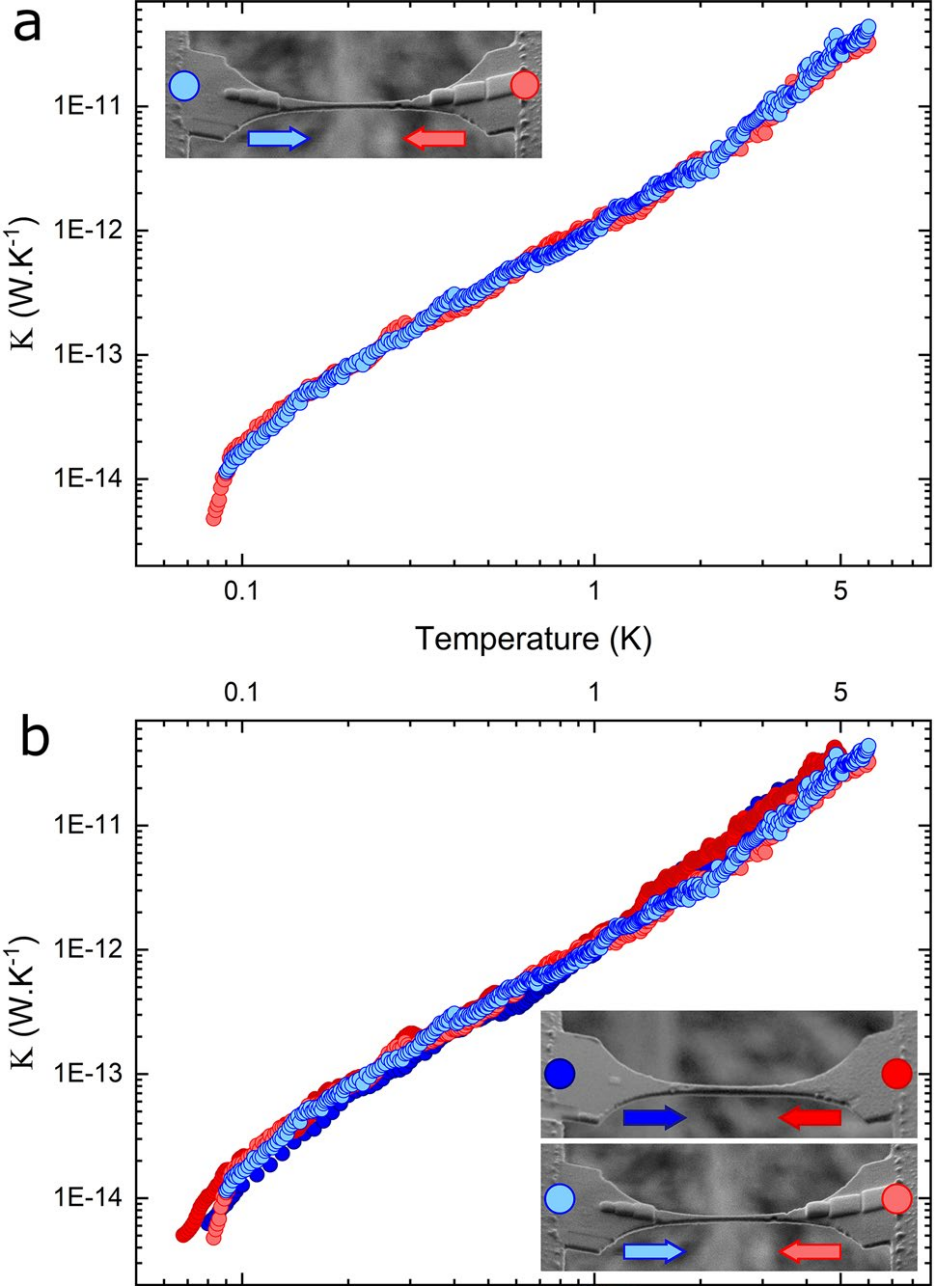
Thermal conductance of nanobeam with asymmetric mass gradient. (**a**) Thermal conductance of the loaded nanobeam with heat flowing in either direction. (upper inset) SEM micrograph of the nanobeam loaded with asymmetric mass gradients on each side. The dot and arrow of a given color correspond to the heated side and heat flux direction, respectively. (**b**) Reported thermal conductance for both heat flux directions in the non-loaded and loaded cases. (inset) SEM micrographs showing the heated side and heat flux direction.

The leads of the previously measured nanobeam are then loaded with the mass gradients; it is important to highlight that the same nanobeam has been used for both experiments. The mass load is set to be asymmetric as it is this asymmetry, coupled to the non-linearities, that is expected to enable rectification. The structure is the one described in the previous section and the measurement is performed with the same conditions, from $\sim$90 mK to 5 K. We see in Fig. 4a the same situation as for the non-loaded nanobeams, i.e., that the thermal conductance is equal in both directions, which suggests that no thermal rectification occurs in this system. The temperature difference is also kept at similar values as for the non-loaded case. Moreover, Fig. 4b shows that the thermal conductance of the nanobeam remains the same whether its leads are loaded or not.

## Discussion

Let us now discuss the reasons for the absence of thermal rectification in this experimental implementation of the scheme proposed by Chen et al. ^41^. In that theoretical work, extremely high rectification factors above 1000% were obtained based on a number of assumptions about the particles and their interactions. First, the presence of a ballistic spacer was confirmed experimentally and is thus conform to the requirement. The mass of the ballistic spacer however should be between that of the light and heavy leads for optimal thermal rectification. In our implementation, the spacer’s mass is the lowest, as both leads are covered by additional material and are thus heavier, with the material deposited on the right lead being $\sim$2 times heavier than on the left lead. These two factors have an extremely strong impact on the rectification factor, as shown by Chen et al. We thus expect that to observe rectification in an experimental implementation of this scheme, a much higher mass asymmetry is required. One such possibility is to deposit much thicker squares of a denser material on one of the leads, albeit this complexifies the fabrication process. Another important factor is the temperature gradient. In our experiment, the temperature difference between both leads is on the order of $\bigtriangleup$T = 20 mK when the experiment is performed at a temperature above 1 K, which corresponds to a temperature gradient $\bigtriangleup$ T/T < 2 %. A higher temperature gradient will be beneficial to increasing the thermal rectification ratio. The last point to consider concerns the non-linearities. In their numerical investigation, Chen et al. assume strongly non-linear interactions. The asymmetry then leads to shifted phonon energy spectra in both leads and the rectification stems from the different overlaps of these phonon energy spectra in both directions of the heat flux. In our fabricated structure, we surmise that the masses loaded on both leads are too small, which combined with a small temperature gradient, do not provide large enough non-linearities to conclusively observe thermal rectification. The causes exposed here as the most plausible reasons for the absence of thermal rectification in our experimental implementation constitute the main differences between the numerical implementation by Chen et al. and this work. Indeed, the design attempted here is adapted to accommodate current fabrication technology limitations, which may explain the absence of rectification. Identifying the precise reason for that result will require further targeted experiments, varying in turn the mass gradient and its spatial distribution, as well as the temperature gradient. It will also be of interest to adapt the numerical model, keeping current fabrication technologies as a prerequisite to the model, to optimize a geometry that can be readily fabricated.

In conclusion, we have reported here the experimental realization and characterization of a loaded nanobeam with asymmetric mass gradient. We have shown that despite the relatively higher density of Pt/C compared to Si$_{3}$N$_{4}$, no thermal rectification was observed. Indeed, several of the conditions required for rectification put forward in the theoretical investigation were not fulfilled strongly enough in this work due to fabrication challenges. We expect that overcoming these challenges should enable the predicted rectification to appear experimentally. Nonetheless, this first experimental attempt at a size-independent thermal rectifier in the microscopic scale gives us clear indications about the required modifications necessary to observe robust thermal rectification. A future implementation will benefit from a higher temperature gradient for which the heaters need to be redesigned, as well as heavier masses, especially on one side, to increase the non-linearities of the system, which is one of the key requirements for thermal rectification. We believe that with these modifications, a robust passive thermal rectifier can be achieved.
